# Rare cavernous hemangioma of adrenal gland: case report

**DOI:** 10.1590/1516-3180.2014.1324715

**Published:** 2014-05-28

**Authors:** Li Wang, Yiwu Dang, Rukun He, Gang Chen

**Affiliations:** I MSc. Postgraduate Student, Department of Pathology, First Affiliated Hospital of Guangxi Medical University, Nanning, Guangxi, China; II MSc. Technician, Department of Pathology, First Affiliated Hospital of Guangxi Medical University, Nanning, Guangxi, China; III MD. Professor, Department of Pathology, First Affiliated Hospital of Guangxi Medical University, Nanning, Guangxi, China; IV MD, PhD. Associate Professor, Department of Pathology, First Affiliated Hospital of Guangxi Medical University, Nanning, Guangxi, China

**Keywords:** Hemangioma, cavernous, Adrenal glands, Hemangioma, Immunohistochemistry, Pathology, clinical, Hemangioma cavernoso, Glândulas suprarrenais, Hemangioma, Imunoistoquímica, Patologia clínica

## Abstract

**CONTEXT:**

Cavernous hemangiomas of the adrenal gland are rare benign neoplastic tumors. The clinical presentation of adrenal hemangiomas is usually vague, and they are often discovered incidentally through imaging examination s performed for other reasons.

**CASE REPORT:**

We report the case of a non-functional adrenal hemangioma found incidentally in a 37-year-old man with a one-year history of headache and hypertension. A right adrenal mass was detected by means of magnetic resonance imaging. Physical examination and all laboratory values were unremarkable. The patient underwent laparoscopic right adrenal gland resection. Histopathological evaluation confirmed adrenal cavernous hemangioma.

**CONCLUSIONS:**

Most occurrences of cavernous hemangiomas of the adrenal gland are non-functional and often discovered incidentally. Although rare, these unusual benign adrenal masses should form part of the differential diagnosis of adrenal neoplasms. The proper treatment for adrenal cavernous hemangioma is surgical removal.

## Abstract (pt)

**CONTEXTO:**

Hemangiomas cavernosos da glândula adrenal são tumores neoplásicos raros, benignos. A apresentação clínica dos hemangiomas adrenais é geralmente vaga e muitas vezes eles são descobertos acidentalmente por exame imagiológico realizado por outras razões.

**RELATO DE CASO:**

Nós relatamos um caso de hemangioma adrenal não funcional encontrado por acaso em um homem de 37 anos de idade com histórico de um ano de dor de cabeça e hipertensão. Foi detectada massa adrenal direita por meio de ressonância magnética. Exame físico e todos os valores de laboratoriais estavam normais. O paciente foi submetido a cirurgia laparoscópica para ressecção da glândula adrenal direita. Avaliação histopatológica confirmou hemangioma adrenal cavernoso.

**CONCLUSÃO:**

A maioria dos hemangiomas cavernosos de glândula adrenal são não funcionais e muitas vezes descobertos por acaso. Embora raras, essas massas adrenais benignas devem ser parte de um diagnóstico diferencial de neoplasias suprarrenais. O tratamento adequado para o hemangioma adrenal cavernoso é a remoção cirúrgica.

## INTRODUCTION

Adrenal hemangiomas are extremely rare. Since the first case in 1955, 63 cases of adrenal hemangioma have been reported, as seen through searching the literature. The clinical presentation of adrenal hemangiomas is usually vague, and non-specific abdominal pain is the predominant symptom.^1^ ^-^ ^3^ Frequently, they are discovered incidentally either during imaging or in autopsies. The development of various diagnostic tools, such as computed tomography and magnetic resonance imaging make it feasible to predict a tumor's nature more precisely.^4^ We report the case of a non-functional adrenal hemangioma found incidentally in a 37-year-old man.

## CASE REPORT

**Figure 1:**
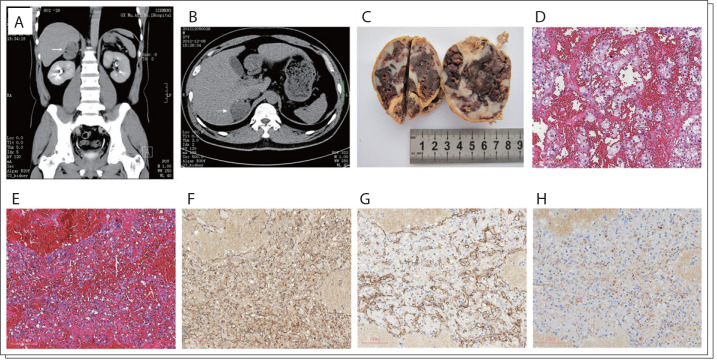
A case of cavernous hemangioma of the adrenal gland. A, B: Computed tomography scan (right adrenal mass, arrows). C: Gross pathology of adrenal mass. D, E: Hematoxylin and eosin staining (× 40). F, G, H: Immunohistochemistry for CD31, CD34 and blood coagulation factor VIII (diaminobenzidine staining, × 40).

A 37-year-old Chinese male was referred to our teaching hospital on December 4th, 2012, with an incidental finding of a right adrenal mass by magnetic resonance imaging. The patient had a one-year history of headache and hypertension. On admission, all vital signs were normal, except blood pressure, reaching 145/101 mmHg. No abnormalities were found in physical examination. All laboratory values were within the normal range. His endocrinology tests were performed in such a way that they would rule out a functional tumor: all the parameters were normal. There were no clinical signs of Cushing's disease or adrenogenital syndrome. An abdominal computed tomography scan revealed a well-defined, heterogeneous, ovoid mass with fat component and peripheral speckled calcifications, which measured 5.3 × 4.6 × 6 cm, located at the upper pole of the right kidney (Figures 1A and 1B). Contrast-enhanced computed tomography showed slight enhancement of the solid component of the mass in the portal venous phase. On December 12, 2012, the patient underwent laparoscopic right adrenal gland resection because the possibility of malignant tumors could not be ruled out clinically. Pathological examination revealed a smoothly and completely encapsulated and moderately firm oval mass, measuring 6 × 5 × 4.5 cm and weighing 150 g, with a cross-section of reddish-brown and ash-gray organized hematoma (Figure 1C). The opened mass contained some areas of necrosis, and the tissue was extremely heterogeneous. Histopathological evaluation showed a cavernous hemangioma with erythrocytes filling the lacunae, which was lined with a single layer of endothelial cells (Figures 1D and 1E). Areas of hemorrhage and small focal calcifications were observed. Atrophy of the adrenal cortex was present under the tumor capsule. There was no evidence of malignancy. Immunohistochemical examination revealed vessels lined with vascular endothelial cells that were specifically positive for CD31, CD34 and blood coagulation factor VIII (Figures 1F, 1G and 1H), demonstrating their endothelial nature. On December 17, 2012, the patient was discharged from the hospital.

## DISCUSSION

Adrenal hemangiomas occur very infrequently. Since the first case in 1955, only 63 cases of adrenal hemangioma have been reported in different databases (Table 1). Adrenal hemangiomas are benign tumors that arise from endothelial cells that line blood vessels. They consist of multiple, large vascular channels lined by a single layer of endothelial cells and supported by collagenous walls. The cause of adrenal hemangioma is not completely understood. Hemangiomas are probably congenital, and hereditary factors may play a role in their pathogenesis. Ectasia, rather than growth, is believed to contribute to hemangioma enlargement.^2^ Adrenal hemangiomas are mostly cavernous, unilateral lesions of the adrenal glands, which appear between the ages of 50 and 70 years, with a 2:1 female-to-male ratio.1,5-6 Our patient was younger than the average age reported. However, adrenal hemangioma in a 19-yearold Saudi female was reported in 2011.^5^ The tumor size has ranged from 2 cm to 25 cm in diameter, and the weight has ranged from a few grams to 5 kg. The majority have measured more than 10 cm, probably because most of these tumors are incidental findings and are usually asymptomatic, unless pain is caused by hemorrhage or mechanical mass effects of the tumor on associated structures.4-6 The size in our case was 6 × 5 × 4.5 cm and the weight was 150 g, i.e. consistent with the range reported.

**Table 1:** Review of the literature on adrenal cavernous hemangioma

Electronic databases	Search strategies	Results	
		Found	Related
PubMed	(adrenal glands neoplasms) OR (adrenal glands) OR (adrenal gland) AND (hemangioma, cavernous)	73	46
Embase	(adrenal glands neoplasms) AND (hemangioma, cavernous)	14	12
Lilacs	(adrenal glands neoplasms) AND (hemangioma) OR (cavernous hemangioma)	38	11

Adrenal hemangiomas are most commonly non-functional tumors, and only three cases of hormone-secreting adrenal hemangiomas have been reported to date.^7^ ^-^ ^9^ These unusual benign adrenal masses are often discovered incidentally during imaging studies performed for other reasons. Because they do not show any symptoms of adrenal hemangiomas, they are frequently diagnosed clinically after reaching a size of 10 cm in diameter. This was similarly observed in our patient, with no associated symptoms of either adrenalism or hypoadrenalism.

Computed tomography and magnetic resonance imaging are the most frequently used methods for diagnosing and characterizing adrenal masses. The typical findings of adrenal hemangioma on computed tomography include a heterogeneous, hypodense lesion with calcifications, as was seen in our patient. Calcification characteristically appears speckled throughout the entire mass. However, it may be difficult to distinguish these lesions from carcinomas or cysts. Magnetic resonance imaging may also show homogeneous masses with central hyperintense signal on T1-weighted images and heterogeneous masses with hyperintense signal on T2-weighted images.^1^ The major tumors that should be differentiated are renal tumors, right-lobe liver tumors and other types of adrenal tumors, as well as the metastases of other carcinomas: melanomas and lung, breast, renal and gastrointestinal cancers.^6^

These lesions are typically well encapsulated and located in the adrenal cortex. On histopathological inspection, most of the tumors reported were cavernous and rarely capillary in type, and they may undergo degenerative changes like thrombosis, hemorrhage, necrosis and calcification. Cavernous hemangiomas are enlarged masses of blood-filled sinusoidal channels that have eroded and displaced normal tissues. Furthermore, the presence of multiple vascular cavities at the periphery is an important feature, which accounts for the characteristic peripheral nodular contrast enhancement pattern seen on imaging.^7^

Surgical resection remains necessary for larger adrenal masses exceeding 3.5 cm,^10^ even when suspected to be of an angiomatous nature, due to their propensity to bleed and the inability to rule out malignant elements. Several open techniques have been described, including transabdominal, flank and posterior approaches. While laparoscopic adrenalectomy has become the procedure of choice for adrenal masses, the majority of these lesions tend to be small. Although it has been reported that adrenalectomy can be performed by means of laparoscopy for lesions measuring less than 6 cm,^6^ laparoscopic resection for larger adrenal hemangiomas of up to 12 cm in diameter has been reported.^2^

## CONCLUSION

In summary, we reported a case of non-functional adrenal cavernous hemangioma discovered by imaging studies. Although rare, cavernous hemangiomas of the adrenal gland should be part of the differential diagnosis for adrenal neoplasms. The proper treatment for adrenal cavernous hemangioma is surgical removal.
